# Plasma 4-Hydroxyproline Levels Are Associated with Diabetes in Chinese Adults: A Cross-Sectional Analysis

**DOI:** 10.3390/metabo16070467

**Published:** 2026-07-04

**Authors:** Qiaoliang Huang, Yingjun Mu, Ruirui Ma, Jiayao Zhu, Xudong Wang, Qingyao Wang, Junyao Huang, Hui Zuo, Jinming Yu

**Affiliations:** 1 School of Public Health, Fudan University, No. 130 Dong’an Road, Shanghai 200032, China; rs10suzhou@163.com; 2 Department of Health Education, Suzhou Center for Disease Control and Prevention, Suzhou 215131, China; 3 School of Public Health, Suzhou Medical College of Soochow University, 199 Ren’ai Rd., Suzhou 215123, China; 20234047007@stu.suda.edu.cn (Y.M.);; 4 Department of Clinical Nutrition, The Fourth Affiliated Hospital of Soochow University (Medical Center of Soochow University), Suzhou 215000, China; 5 Jiangsu Key Laboratory of Preventive and Translational Medicine for Major Chronic Non-Communicable Diseases, Suzhou 215123, China; 6 MOE Key Laboratory of Geriatric Diseases and Immunology, Suzhou Medical College of Soochow University, Suzhou 215123, China

**Keywords:** proline metabolism, 4-hydroxyproline, diabetes, metabolomics introduction

## Abstract

Background: 4-Hydroxyproline, a product of collagen turnover generated through prolyl hydroxylation, has been implicated in metabolic regulation. While previous studies have associated circulating proline with diabetes, the relationship between 4-hydroxyproline and diabetes remains unclear. Methods: We conducted a cross-sectional analysis of 796 adults aged 35–74 years from Changshu, eastern China. Plasma 4-hydroxyproline levels were quantified using ultra-high-performance liquid chromatography–tandem mass spectrometry. Unconditional logistic regression was used to estimate odds ratios (ORs) and 95% confidence intervals (CIs). Results: Higher plasma 4-hydroxyproline levels were associated with increased odds of diabetes after multivariable adjustment (per SD increase: OR = 1.35, 95% CI: 1.10–1.66; *p* = 0.004). Participants in the highest quartile had higher odds of diabetes than those in the lowest quartile (Q4 vs. Q1: OR = 1.86, 95% CI: 1.01–3.47; *p* for trend = 0.006). The associations remained materially unchanged after excluding insulin users, individuals with diabetes-related complications, and those using antidiabetic medication. Further adjustment for dietary intake and proline did not materially alter the results. Conclusions: Higher plasma 4-hydroxyproline levels were associated with diabetes. Given the cross-sectional design, causality cannot be inferred, and reverse causation cannot be excluded. Prospective studies are needed to confirm these findings.

## 1. Introduction

Diabetes is one of the leading causes of death and disability worldwide [1]. China has the largest population of individuals with diabetes, with recent estimates indicating that more than 118 million adults are affected [2]. The economic burden of diabetes on the Chinese healthcare system is substantial, with direct costs projected to rise from US$190.2 billion in 2020 to US$337.8 billion in 2030 [2,3]. The prevalence of comorbidities and complications is also persistently high among patients with diabetes [4]. Therefore, it is essential to identify novel risk factors and underlying mechanisms to improve diabetes prevention and disease management.

4-Hydroxyproline is a major collagen-specific amino acid that is critical for structural integrity in animals. It is generated by prolyl 4-hydroxylase through the hydroxylation of proline residues in collagen-related proteins [5,6]. Inside cells, it can be metabolized by mitochondrial hydroxyproline oxidase (OH-POX) or released into the extracellular space and subsequently enter the bloodstream. When mitochondrial OH-POX oxidizes hydroxyproline, it produces reactive oxygen species (ROS) [7,8]. In addition, 4-hydroxyproline can scavenge oxidants and suppress NF-κB activation. It may also help regulate intracellular redox homeostasis and stimulate the expression of antioxidant enzymes [9].

A previous study reported reduced levels of hydroxyproline in patients with diabetes [10]. Additionally, higher 4-hydroxyproline levels have been linked to reduced endothelial injury in patients with type 2 diabetes (T2D) and may reflect an adaptive response to oxidative stress [11]. Elevated vitreous hydroxyproline in proliferative diabetic retinopathy (PDR) may promote adaptogenic changes in retinal pericytes [12]. In contrast, diabetic Goto-Kakizaki rats exposed to exogenous Nε-(carboxymethyl)lysine showed altered 4-hydroxyproline levels alongside changes in oxidative stress and amino acid, tricarboxylic acid (TCA) cycle, and carbohydrate metabolism, which may be related to diabetic complications, including nephropathy, retinopathy, and impaired wound healing [13]. However, evidence on the association between 4-hydroxyproline and diabetes in the general population remains limited. Therefore, we conducted a cross-sectional study to investigate the association between plasma 4-hydroxyproline levels and diabetes in a community-based population.

## 2. Materials and Methods

### 2.1. Study Subjects

This cross-sectional study included adults aged 35–74 years from Changshu, eastern China. Eligible participants were free of stroke at baseline, agreed to participate, and provided written informed consent. Individuals with malignant tumors, severe disability (unable to independently complete the questionnaire survey or physical measurements), or psychiatric disorders at baseline were excluded [14,15].

Demographic characteristics, lifestyle factors, diet, medication use, and family history of disease were collected by trained staff using standardized questionnaires. A total of 796 participants were included in the current cross-sectional analysis [16]. Informed consent was obtained from each participant, and the study was approved by the Ethics Review Committee of Soochow University, Suzhou, China.

### 2.2. Laboratory Measurements

Venous blood was collected at baseline from participants after an overnight fast of at least 8 h and placed in EDTA tubes. Plasma was then separated and stored at −80 °C in the biobank of the School of Public Health of Soochow University until analysis. Metabolite levels were quantitatively measured using ultra-high-performance liquid chromatography–tandem mass spectrometry (UHPLC-MS/MS). Plasma proline, 4-hydroxyproline, and cotinine (a biomarker of recent nicotine exposure) were quantified using an Agilent 1290 Infinity II UHPLC system coupled with a 6470A Triple Quadrupole mass spectrometer (UHPLC-MS/MS, 1290 Infinity II/6470A, Agilent Technologies Inc., Santa Clara, CA, USA). Frozen plasma was thawed, and 20 μL of sample was mixed with stable isotope-labeled internal standards, followed by the addition of 120 μL methanol and 10 μL dithiothreitol (0.5 M in water). The mixture was vortexed for 1 min, incubated at 25 °C for 30 min, kept at −20 °C for 30 min, and centrifuged at 15,000 *g* and 4 °C for 15 min. Then, 20 μL of supernatant was mixed with 30 μL of 100 mM sodium carbonate and 50 μL of 2% benzoyl chloride in acetonitrile for derivatization at 25 °C for 10 min. Samples were injected onto a Waters UPLC BEH C18 column (100 mm × 2.1 mm, 1.7 μm; Waters Corporation, Milford, MA, USA) at 0.25 mL/min. The mobile phases consisted of water containing 10 mM ammonium formate and 0.15% formic acid (A) and acetonitrile (B). The column temperature was set at 40 °C. ESI+ ionization was used in positive mode, with drying gas at 300 °C, sheath gas at 350 °C, and flow rates of 5 and 11 L/min, respectively. The nebulizer pressure was 40 psi, and the capillary voltage was 4000 V. The retention times were 6.36 min for proline and 3.76 min for 4-hydroxyproline. The coefficients of determination (R^2^) were 0.99799 for proline and 0.99919 for 4-hydroxyproline. Calibration curves were prepared from serial dilutions of analytical standards after benzoyl chloride derivatization. Pooled quality-control samples were prepared from randomly selected samples and included in each batch. The assay showed good precision, with within-day coefficients of variation (CVs) ranging from 0.62% to 0.79% and between-day CVs from 9.32% to 14.77%. All samples were analyzed using a standardized protocol to minimize potential batch effects.

Plasma glucose, total cholesterol (TC), triglycerides (TG), high-density lipoprotein cholesterol (HDL-C), and creatinine levels were also measured [15]. The estimated glomerular filtration rate (eGFR) was calculated using the Chronic Kidney Disease Epidemiology Collaboration equation [17].

### 2.3. Definition of Diabetes and Diabetes Complications

Information on diabetes was collected at baseline. Diabetes was defined as a fasting plasma glucose level ≥ 7.0 mmol/L (N = 96), a self-reported physician diagnosis of diabetes (N = 62), or current use of antidiabetic medication (N = 45) [16]. A total of 36 participants met more than one diabetes criterion. Diabetes complications were defined as self-reported physician-diagnosed diabetic retinopathy or diabetic nephropathy.

### 2.4. Covariates

Body mass index (BMI) was calculated as weight in kilograms divided by the square of height in meters (kg/m^2^). Educational attainment was stratified into three categories: 0 years (no formal school education), 1–5 years (primary school), and ≥6 years (middle school, college, or higher education). Smoking status was determined based on self-report, and those self-reported nonsmoking participants with cotinine levels ≥ 85 nmol/L were reclassified as current smokers [18]. Alcohol consumption was assessed through self-report; individuals who consumed ≥12 alcoholic drinks in the past 12 months were classified as drinkers [19]. Hypertension was defined by self-report, systolic blood pressure ≥ 140 mmHg, diastolic blood pressure ≥ 90 mmHg, or current use of antihypertensive medications. Dyslipidemia was defined as self-reported use of lipid-lowering treatment or abnormal laboratory measurements (TC > 6.20 mmol/L, TG > 2.30 mmol/L, or HDL-C < 1.00 mmol/L). Physical activity was assessed using the Global Physical Activity Questionnaire and expressed as metabolic equivalent-hours per day (MET-h/d).

### 2.5. Statistical Analyses

Demographic and clinical characteristics are summarized as medians (interquartile ranges), while categorical variables are presented as numbers (percentages). The Wilcoxon rank-sum test was used to compare continuous variables, and the chi-square test was applied to compare categorical variables between the two groups. Plasma 4-hydroxyproline was log-transformed to better approximate a normal distribution. Quartiles of metabolite concentrations were defined based on the distribution of the study population. Unconditional logistic regression models were used to estimate odds ratios (ORs) and 95% confidence intervals (CIs). ORs and 95% CIs were calculated per standard deviation (SD) increase in the log-transformed metabolite and across quartiles of metabolite levels, with additional analyses excluding individuals with severe diabetes (defined as insulin use or the presence of diabetic retinopathy or nephropathy). Multivariable models were adjusted for age, sex, BMI, educational attainment, current smoking status, alcohol consumption, hypertension, dyslipidemia, estimated glomerular filtration rate (eGFR), and physical activity.

In stratified analyses, unconditional logistic regression models and interaction tests were conducted to explore possible effect modification on the associations. Additionally, restricted cubic spline models were used to examine the potential nonlinear relationship between 4-hydroxyproline and diabetes.

Sensitivity analyses were conducted to assess the robustness of the findings by further adjustment for (1) red meat intake, (2) total protein intake, (3) total energy intake, and (4) proline levels, based on the multivariable-adjusted models. All statistical analyses were conducted using R (version 4.3.2). All tests were two-sided, and a *p*-value of <0.05 was considered statistically significant. Model assumptions were evaluated as follows: linearity of continuous covariates was assessed using the Box–Tidwell test, and multicollinearity was examined using variance inflation factors (VIFs), with all values < 2 indicating no meaningful collinearity.

## 3. Results

### 3.1. Participant Characteristics

The baseline demographic and biochemical characteristics of the 796 participants are presented in Table 1. Among these participants, 114 had diabetes. Individuals with diabetes had a higher BMI, a higher prevalence of dyslipidemia, and higher levels of proline and 4-hydroxyproline compared with participants without diabetes (*p* < 0.05).

### 3.2. Association Between 4-Hydroxyproline and Diabetes

**Figure 1:**
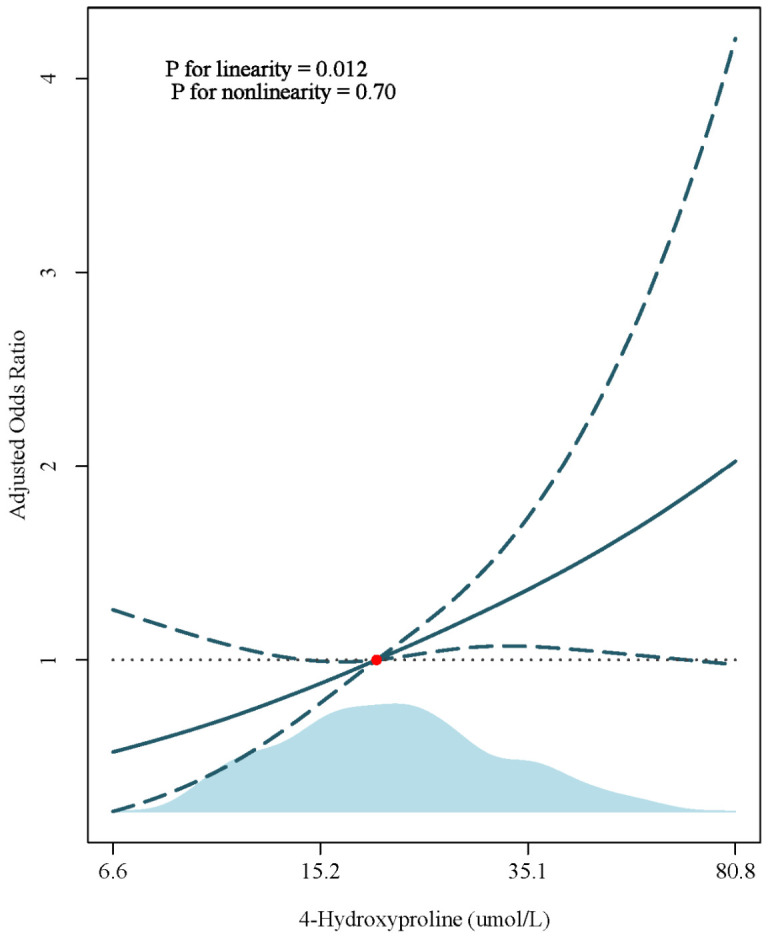
Multivariable-adjusted association of 4-hydroxyproline with diabetes by restricted cubic spline regression. The blue shaded area indicates the distribution of 4-hydroxyproline. The blue solid line shows odds ratio, and the blue dotted lines represent the 95% confidence interval. The red dot represent the inflection point. Restricted cubic spline with three knots located at the 10th, 50th, and 90th percentiles of 4-hydroxyproline. Estimates were adjusted for age (years, continuous); sex (female, male), body mass index (<18.5 kg/m^2^, 18.5–23.9 kg/m^2^, 24–27.9 kg/m^2^, ≥28 kg/m^2^), educational attainment (0 year, 1–5 years, or ≥6 years), current smoking (yes or no), drinking (yes or no), hypertension (yes or no), hyperlipidemia (yes or no), estimated glomerular filtration rate (<60 mL/min/1.73 m^2^, ≥ 60 mL/min/1.73 m^2^), and physical activity (MET-h/d, continuous).

As shown in Table 2, after adjustment for age, sex, BMI, educational attainment, current smoking, alcohol consumption, hypertension, dyslipidemia, eGFR, and physical activity, each 1-SD increment in log-transformed 4-hydroxyproline was generally positive association with diabetes (OR = 1.35, 95% CI: 1.10–1.66; *p* = 0.004). Compared with participants in the lowest quartile, those in the highest quartile had higher odds of diabetes (OR = 1.86, 95% CI: 1.01–3.47; *p* for trend = 0.006). These associations remained essentially unchanged after excluding insulin users (per SD: OR = 1.35, 95% CI: 1.09–1.66; *p* = 0.005; Q4 vs. Q1: OR = 1.80, 95% CI: 0.97–3.43; *p* for trend = 0.007), individuals with diabetes-related complications (per SD: OR = 1.37, 95% CI: 1.11–1.70; *p* = 0.004; Q4 vs. Q1: OR = 1.96, 95% CI: 1.03–3.80; *p* for trend = 0.007) or antidiabetic medication users (per SD: OR =1.35, 95% CI: 1.05–1.73; *p* = 0.020; Q4 vs. Q1: OR = 1.96, 95% CI: 0.90–4.40; *p* for trend = 0.024). As shown in Figure 1, the multivariable-adjusted restricted cubic spline model suggested a generally positive association between plasma 4-hydroxyproline levels and diabetes (*p* for linearity = 0.012).

### 3.3. Stratified Analyses and Sensitivity Analyses

**Figure 2:**
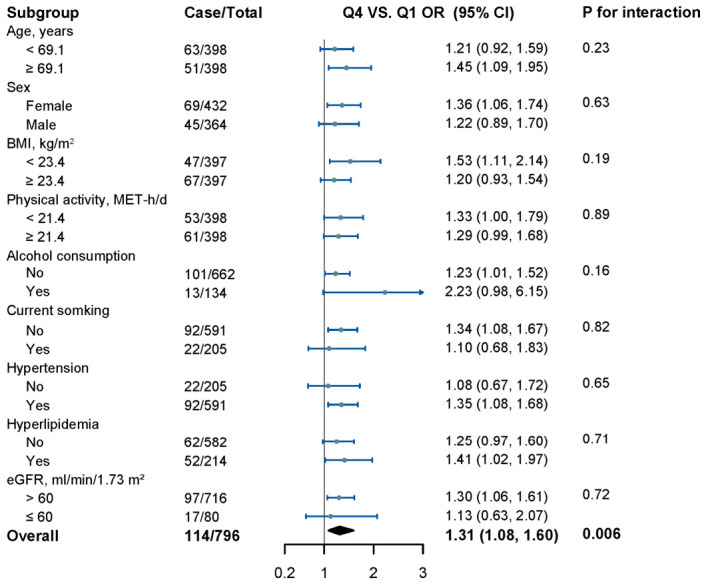
Stratified analyses of the association between plasma 4-hydroxyproline and diabetes by unconditional logistic regression. All models were adjusted for age (years, continuous), sex (female, male), body mass index (<18.5 kg/m^2^, 18.5–23.9 kg/m^2^, 24–27.9 kg/m^2^, ≥28 kg/m^2^), educational attainment (0 year, 1–5 years, or ≥6 years), current smoking (yes or no), drinking (yes or no), hypertension (yes or no), hyperlipidemia (yes or no), estimated glomerular filtration rate (<60 mL/min/1.73 m^2^, ≥60 mL/min/1.73 m^2^), and physical activity (MET-h/d, continuous).

Subgroup analyses were conducted according to age, sex, BMI, educational attainment, smoking status, alcohol consumption, hypertension, dyslipidemia, eGFR, and physical activity. We did not detect statistically significant effect modification across the stratified groups (all *p* for interaction > 0.05) (Figure 2). However, these subgroup analyses were exploratory and may have been underpowered because only 114 participants had diabetes.

Notably, the associations remained materially unchanged after further adjustment for red meat intake, total protein intake, total energy intake, or proline (Table 3). In addition, proline was positively associated with diabetes after additional adjustment for insulin users, and individuals with diabetes-related complications (Table S1). No evidence of nonlinearity was observed for age (*p* = 0.60) or physical activity (*p* = 0.17), and the global test supported the linearity assumption (*p* = 0.36). Multicollinearity was minimal, with all variance inflation factors (VIFs) < 2; specifically, the VIFs were 1.12 for proline and 1.14 for 4-hydroxyproline.

## 4. Discussion

### 4.1. Principal Findings

In this community-based cross-sectional study, higher plasma 4-hydroxyproline levels were associated with increased odds of diabetes. The observed association remained materially unchanged after excluding individuals using insulin or antidiabetic medications and those with diabetes-related complications. Further adjustment for dietary factors including red meat intake, total protein intake, and total energy intake, and plasma proline did not materially alter the results. Overall, the effect estimates were modest and should be interpreted as hypothesis generating rather than clinically actionable at this stage.

### 4.2. 4-Hydroxyproline and Diabetes

To our knowledge, no previous studies have specifically examined the relationship between plasma 4-hydroxyproline and diabetes. In this study, higher plasma 4-hydroxyproline levels were positively associated with diabetes. Although direct evidence linking 4-hydroxyproline to diabetes is limited, prior metabolomic studies have reported higher plasma proline levels in individuals with elevated insulin concentrations [20]. In addition, cohort studies have identified associations between circulating proline and an increased risk of incident T2D [21,22]. 4-Hydroxyproline is derived from collagen proline residues and reflects collagen turnover; therefore, it may capture metabolic information related to, but distinct from, circulating proline. Given the cross-sectional design, this association should not be interpreted as evidence that 4-hydroxyproline contributes to diabetes development.

However, findings from previous studies have been inconsistent. One study reported that insulin dysregulation was associated with lower trans-4-hydroxyproline levels, but this study was conducted in only 20 horses [23]. The Botnia prospective study further reported that bradykinin hydroxyproline (a bradykinin analog in which the third amino acid, proline, is hydroxylated) was associated with a decreased risk of T2D in a Finnish population [24], while an animal study suggested that proline hydroxylation may have therapeutic potential for hyperglycemia and T2D [25]. These discrepancies may reflect differences in populations, species, biospecimens, metabolite forms, and study designs. In contrast, studies in animal models of diabetes have reported elevated hydroxyproline levels, reflecting enhanced proline hydroxylation and collagen accumulation that may contribute to diabetic complications such as cytopathy [26]. To our knowledge, this is the first study to report a positive association between plasma 4-hydroxyproline levels and diabetes. Notably, reverse causation cannot be excluded. As the biochemical precursor of 4-hydroxyproline, proline may lie on the causal pathway. The attenuation after additional adjustment for proline suggests that 4-hydroxyproline may partly reflect broader alterations in proline metabolism or collagen turnover. Because proline is a precursor of 4-hydroxyproline and may lie on the same biological pathway, this adjustment may also represent overadjustment. Therefore, the proline-adjusted results should be interpreted cautiously. Further large prospective studies are warranted to clarify temporality and to determine related amino acid metabolites.

### 4.3. Possible Mechanisms

Several biological processes may help explain the observed association. However, given the cross-sectional design, these mechanisms are hypothesis-generating, do not imply causality, and are not proven by the current study. First, proline has been shown to reduce paraspeckle formation in hepatocytes, which leads to the release of retained mRNA species, including Ppargc1a and Foxo1, into the cytoplasm for protein synthesis. These transcripts subsequently promote hepatic gluconeogenesis, which may contribute to hyperglycemia [27]. Second, prolyl 4-hydroxylases (P4Hs), key enzymes in collagen synthesis and oxygen sensing, have been implicated in metabolic regulation, and modulation of hypoxia-inducible factor (HIF) pathways has demonstrated metabolic effects in experimental diabetes [28,29,30]. Notably, hyperglycemia may impair HIF-1α stability and transcriptional activity, thereby influencing P4H function and downstream prolyl hydroxylation processes, including the generation of 4-hydroxyproline [31,32]. Third, prolyl hydroxylase domain protein 3 (PHD3) regulates hepatic gluconeogenesis via post-translational modification of CREB-regulated transcriptional coactivator 2 (CRTC2), thereby influencing glucose homeostasis [25]. Importantly, 4-hydroxyproline is predominantly derived from collagen degradation and may serve as a marker of extracellular matrix turnover rather than a direct indicator of intracellular enzymatic activity [33]. Elevated 4-hydroxyproline may reflect downstream effects of diabetes, including alterations in extracellular matrix turnover, renal function, and metabolic processes, rather than serving as a causal factor [34,35].

### 4.4. Strengths and Limitations

The present study provides a systematic evaluation of plasma 4-hydroxyproline, a biomarker of collagen turnover and prolyl hydroxylation, in relation to diabetes in a community-based population. The use of standardized measurements, adjustment for multiple demographic, lifestyle, and dietary factors, and consistent findings across sensitivity analyses support the robustness of the results. First, the cross-sectional nature of this secondary analysis and the lack of longitudinal follow-up preclude causal inference. Therefore, we cannot determine whether higher 4-hydroxyproline contributes to diabetes, results from diabetes-related tissue remodeling, or reflects altered collagen turnover secondary to hyperglycemia; reverse causation cannot be excluded. In addition, selection bias may limit the generalizability of the findings to the underlying population, although it is unlikely to substantially bias the cross-sectional associations based on baseline measurements. Second, dietary intake was assessed using a simplified semi-quantitative food frequency questionnaire and is subject to measurement error and recall bias, which may result in underestimation of actual intake. Third, diabetes was defined using a single fasting glucose measurement and without HbA1c or OGTT, which may have led to misclassification, particularly because undiagnosed diabetes or impaired glucose regulation may have been missed. Such misclassification is likely to be non-differential and would generally attenuate the observed association. Additionally, information on diabetes complications was limited to self-reported physician-diagnosed diabetic retinopathy and diabetic nephropathy. Fourth, although the quartile-based associations were not strictly monotonic, the continuous and spline analyses supported an overall positive linear association. This pattern may reflect residual confounding, or statistical instability due to the limited number of diabetes cases. Fifth, residual confounding remains possible. Although we adjusted for eGFR and several metabolic and lifestyle factors, information on inflammatory markers, renal function, liver function, detailed collagen turnover markers, dietary collagen intake, physical frailty, collagen-related disease, osteoporosis, related medication use, protein supplementation, and chronic inflammatory conditions was not available. Additionally, although blood samples were collected after an overnight fast, information on antidiabetic medication type, medication timing, diabetes duration, and glycemic control was unavailable. These factors may influence circulating 4-hydroxyproline levels and should be considered in future studies. Sixth, 4-hydroxyproline was measured at a single time point; within-person variability may have introduced non-differential measurement error, likely biasing estimates toward the null. Finally, the spline estimates at the extremes of 4-hydroxyproline concentrations had wider confidence intervals, likely because fewer participants were in those ranges. In addition, the study population was limited to community-dwelling Chinese adults from one region, which may limit generalizability.

## 5. Conclusions

Higher plasma 4-hydroxyproline levels were associated with diabetes in this cross-sectional analysis. Given the study design, causality cannot be inferred, and reverse causation remains possible. It may reflect diabetes-related collagen turnover, extracellular matrix remodeling, renal or metabolic alterations, or tissue damage rather than a contributor to diabetes development. Given the high burden of diabetes in China, identifying diabetes-related metabolic markers may help improve understanding of metabolic alterations associated with diabetes and support future biomarker discovery and metabolic risk stratification. Prospective studies are needed to clarify causality, temporal relationships, and underlying mechanisms.

## Figures and Tables

**Table 1:** Demographic and biochemical characteristics of the participants. Abbreviations: BMI, body mass index; eGFR, estimated glomerular filtration rate. ^1^ Adjusted for total energy intake by using the residual method. Differences between participants with and without diabetes were compared by chi-square tests. The continuous variables are expressed as median (interquartile range) and categorical variables as number (n) and percentage (%). *p*-Values were calculated by Wilcoxon test for continuous variables and chi-square test for categorical variables.

	Overall (n = 796)	Diabetes		*p*-Value
		No (n = 682)	Yes (n = 114)	
Age, years	69.1 (62.6, 75.1)	69.3 (62.8, 75.3)	68.4 (61.4, 73.3)	0.07
Sex (%)				
Male	364 (45.7)	319 (46.8)	45 (39.5)	0.18
Female	432 (54.3)	363 (53.2)	69 (60.5)	
Current smoking (%)				
No	591 (74.2)	499 (73.2)	92 (80.7)	0.11
Yes	205 (25.8)	183 (26.8)	22 (19.3)	
Drinking (%)				
No	662 (83.2)	561 (82.3)	101 (88.6)	0.12
Yes	134 (16.8)	121 (17.7)	13 (11.4)	
Dyslipidemia (%)				
No	582 (73.1)	520 (76.2)	62 (54.4)	<0.001
Yes	214 (26.9)	162 (23.8)	52 (45.6)	
Hypertension (%)				
No	205 (25.8)	183 (26.8)	22 (19.3)	0.11
Yes	591 (74.2)	499 (73.2)	92 (80.7)	
BMI, kg/m^2^	23.0 (21.3, 25.8)	23.3 (21.1, 25.5)	24.4 (21.9, 27.0)	0.001
Physical activity, MET-h/d	21.4 (12.9, 34.9)	21.1 (12.9, 34.8)	22.4 (12.6, 35.4)	0.86
Animal food intake (g/d) ^1^				
Red meat intake	25.0 (13.5, 41.0)	24.9 (13.3, 40.4)	27.7 (14.1, 43.0)	0.33
Poultry intake	17.0 (7.4, 30.2)	17.0 (7.0, 30.2)	16.8 (9.9, 30.1)	0.63
Fish/seafood intake	26.3 (14.0, 44.8)	26.3 (14.2, 44.7)	26.0 (12.9, 45.9)	0.79
Egg intake	22.4 (12.2, 41.5)	22.0 (12.2, 42.2)	24.6 (12.3, 39.9)	0.81
Total	108.3 (67.6, 167.7)	107.9 (67.6, 165.9)	112.8 (66.8, 193.3)	0.33
Total protein intake (g/d) ^1^	55.1 (50.1, 63.0)	55.0 (50.3, 62.8)	55.7 (49.6, 64.2)	0.79
Total energy intake (kcal/d)	1872.3 (1473.0, 2247.7)	1889.6 (1474.7, 2273.1)	1774.0 (1444.7, 2113.1)	0.26
Antidiabetic medication use (%)				<0.001
No	751 (94.3)	682 (100)	69 (60.5)	
Yes	45 (5.7)	0 (0.0)	45 (39.5)	
Self-report diabetes (%)				<0.001
No	645 (91.2)	601 (100.0)	44 (41.5)	
Yes	62 (8.8)	0 (0.0)	62 (58.5)	
Fasting glucose (%)				<0.001
<7.0 mmol/L	700 (87.9)	682 (100.0)	18 (15.8)	
≥7.0 mmol/L	96 (12.1)	0 (0.0)	96 (84.2)	
eGFR, ml/min/1.73 m^2^				
<60	716 (89.9)	619 (90.8)	97 (85.1)	0.09
≥60	80 (10.1)	63 (9.2)	17 (14.9)	
Proline (umol/L)	213.3 (173.7, 261.9)	208.1 (172.4, 255.9)	229.0 (183.3, 300.8)	0.001
4-Hydroxyproline (umol/L)	17.9 (12.3, 27.0)	17.2 (12.1, 26.3)	21.7 (14.6, 33.0)	0.010

**Table 2:** Odds ratios (ORs) and 95% confidence intervals (CIs) for diabetes by unconditional logistic regression (n = 796).

4-Hydroxyproline, umol/L	Quartile (Q)				*p* for Trend ^a^	Continuous ^b^	*p*-Value ^b^
	Q1	Q2	Q3	Q4			
Model 1	Ref	0.97 (0.51, 1.84)	1.95 (1.10, 3.53)	1.96 (1.09, 3.58)	0.004	1.37 (1.13, 1.67)	0.002
Model 2	Ref	0.86 (0.44, 1.67)	1.95 (1.08, 3.58)	1.86 (1.01, 3.47)	0.006	1.35 (1.10, 1.66)	0.004
After exclusion of individuals who used insulin (n = 8)							
Model 1	Ref	0.89 (0.45, 1.74)	2.03 (1.13, 3.72)	1.92 (1.05, 3.56)	0.005	1.37 (1.12, 1.68)	0.002
Model 2	Ref	0.77 (0.39, 1.54)	1.99 (1.09, 3.70)	1.80 (0.97, 3.43)	0.007	1.35 (1.09, 1.66)	0.005
After exclusion of individuals with diabetes complications (n = 16)							
Model 1	Ref	0.98 (0.49, 1.94)	1.92 (1.04, 3.62)	2.00 (1.08, 3.81)	0.006	1.38 (1.12, 1.70)	0.002
Model 2	Ref	0.88 (0.43, 1.78)	1.93 (1.03, 3.68)	1.96 (1.03, 3.80)	0.007	1.37 (1.11, 1.70)	0.004
After exclusion of individuals who use antidiabetic medication (n = 45)							
Model 1	Ref	1.08 (0.47, 2.49)	2.21 (1.07, 4.79)	2.01 (0.95, 4.44)	0.016	1.35 (1.06, 1.72)	0.021
Model 2	Ref	0.98 (0.42, 2.30)	2.16 (1.03, 4.74)	1.96 (0.90, 4.40)	0.024	1.35 (1.05, 1.73)	0.020

Abbreviations: Ref, reference. Model 1: adjusted for age (years, continuous), and sex (male, female). Model 2: further adjusted for body mass index (<18.5 kg/m^2^, 18.5–23.9 kg/m^2^, 24–27.9 kg/m^2^, ≥28 kg/m^2^), educational attainment (0 year, 1–5 years, or ≥6 years), current smoking (yes or no), drinking (yes or no), hypertension (yes or no), dyslipidemia (yes or no), estimated glomerular filtration rate (<60 mL/min/1.73 m^2^, ≥60 mL/min/1.73 m^2^), and physical activity (MET-h/d, continuous). ^a^ *p* trend values were only applied to the associations between categorical variables (Q1–Q4) and diabetes. ^b^ ORs (95% CIs) were reported per one standard deviation increment of log-transformed 4-hydroxyproline.

**Table 3:** Odds ratios (ORs) and 95% confidence intervals (CIs) for diabetes with additional alternatives.

	Quartile (Q)				*p* for Trend ^a^	Continuous ^b^	*p*-Value ^b^
	Q1	Q2	Q3	Q4			
Model 2+ red meat intake ^1^	Ref	0.86 (0.44, 1.67)	1.95 (1.08, 3.58)	1.86 (1.01, 3.47)	0.006	1.35 (1.10, 1.66)	0.004
Model 2+ total protein intake ^2^	Ref	0.86 (0.44, 1.66)	1.95 (1.08, 3.58)	1.85 (1.01, 3.46)	0.006	1.35 (1.10, 1.66)	0.004
Model 2+ total energy intake ^3^	Ref	0.86 (0.44, 1.67)	1.95 (1.08, 3.58)	1.82 (0.99, 3.40)	0.008	1.34 (1.09, 1.65)	0.005
Model 2+ proline ^4^	Ref	0.8 (0.41, 1.55)	1.78 (0.98, 3.29)	1.51 (0.79, 2.92)	0.040	1.27 (1.02, 1.58)	0.036

Abbreviations: Ref, reference. ^1,2^ The model was further adjusted for red meat intake and total protein intake, respectively. ^3^ The model was further adjusted for total energy intake. ^4^ The model was further adjusted for plasma proline. ^a^ *p* trend values were only applied to the associations between categorical variables (Q1–Q4) and diabetes. ^b^ ORs (95% CIs) were reported per one standard deviation increment of log-transformed 4-hydroxyproline.

## Data Availability

The original contributions presented in this study are included in the article/Supplementary Materials. Further inquiries can be directed to the corresponding authors.

## Supplementary Materials

The following supporting information can be downloaded at https://www.mdpi.com/article/10.3390/metabo16070467/s1. Table S1. Odds ratios (ORs) and 95% confidence intervals (CIs) for diabetes by unconditional logistic regression (n = 796).
