# Supplementary material for: Plasma 4-Hydroxyproline Levels Are Associated with Diabetes in Chinese Adults: A Cross-Sectional Analysis
Source: Metabolites. 2026 Jul 4;16(7):467. doi: 10.3390/metabo16070467 (PMC13414147; doi:10.3390/metabo16070467)
Supplement: Supplementary file 1 [file metabolites-16-00467-s001.zip › metabolites-4385613-supplementary.pdf]

**Table S1.** Odds ratios (ORs) and 95% confidence intervals (CIs) for diabetes by unconditional logistic regression (n=796)

| Proline, umol/L | Quartile (Q) | | | | *P* for trend[a] | Continuous[b] | *P* value[b] |
|---|---|---|---|---|---|---|---|
| | Q1 | Q2 | Q3 | Q4 | | | |
| Model 1 | Ref | 0.98 (0.52, 1.85) | 1.57 (0.87, 2.86) | 2.16 (1.23, 3.90) | 0.002 | 1.46 (1.20, 1.78) | <0.001 |
| Model 2 | Ref | 0.83 (0.43, 1.60) | 1.30 (0.71, 2.42) | 1.73 (0.96, 3.20) | 0.022 | 1.37 (1.11, 1.69) | 0.003 |
| After exclusion of individuals who used insulin (n= 8) | | | | | | | |
| Model 1 | Ref | 1.00 (0.52, 1.95) | 1.63 (0.89, 3.04) | 2.31 (1.28, 4.25) | 0.002 | 1.49 (1.21, 1.83) | <0.001 |
| Model 2 | Ref | 0.84 (0.42, 1.67) | 1.34 (0.71, 2.56) | 1.85 (1.00, 3.51) | 0.015 | 1.40 (1.13, 1.74) | 0.002 |
| After exclusion of individuals with diabetes complications (n= 16) | | | | | | | |
| Model 1 | Ref | 0.94 (0.48, 1.84) | 1.42 (0.76, 2.68) | 2.03 (1.12, 3.76) | 0.008 | 1.43 (1.16, 1.76) | <0.001 |
| Model 2 | Ref | 0.82 (0.41, 1.65) | 1.21 (0.63, 2.34) | 1.72 (0.92, 3.27) | 0.036 | 1.37 (1.11, 1.69) | 0.003 |
| After exclusion of individuals who used antidiabetic medication (n= 45) | | | | | | | |
| Model 1 | Ref | 0.88 (0.41, 1.90) | 1.18 (0.57, 2.48) | 1.72 (0.86, 3.52) | 0.08 | 1.38 (1.08, 1.76) | 0.010 |
| Model 2 | Ref | 0.72 (0.32, 1.60) | 1.00 (0.47, 2.17) | 1.35 (0.65, 2.87) | 0.24 | 1.31 (1.01, 1.68) | 0.039 |

Abbreviations: Ref, reference.

Model 1: adjusted for age (years, continuous), and sex (male, female).

Model 2: further adjusted for body mass index (< 18.5 kg/m$^2$, 18.5-23.9 kg/m$^2$, 24-27.9 kg/m$^2$, ≥ 28 kg/m$^2$), educational attainment (0 year, 1-5 years, or ≥ 6 years), current smoking (yes or no), drinking (yes or no), hypertension (yes or no), dyslipidemia (yes or no), estimated glomerular filtration rate (< 60 ml/min/1.73 m$^2$, ≥ 60 ml/min/1.73 m$^2$), and physical activity (MET-h/d, continuous).

[a] P trend values were only applied to the associations between categorical variables (Q1-Q4) and diabetes.

[b] ORs (95% CIs) were reported per one standard deviation increment of log-transformed proline.
